# Brain Activation Time-Locked to Sleep Spindles Associated With Human Cognitive Abilities

**DOI:** 10.3389/fnins.2019.00046

**Published:** 2019-02-06

**Authors:** Zhuo Fang, Laura B. Ray, Adrian M. Owen, Stuart M. Fogel

**Affiliations:** ^1^Brain and Mind Institute, Western University, London, ON, Canada; ^2^School of Psychology, University of Ottawa, Ottawa, ON, Canada; ^3^Sleep Unit, The Royal’s Institute of Mental Health Research, University of Ottawa, Ottawa, ON, Canada; ^4^Department of Psychology, Western University, London, ON, Canada; ^5^University of Ottawa Brain and Mind Research Institute, Ottawa, ON, Canada

**Keywords:** sleep, spindles, cognitive abilities, simultaneous EEG–fMRI, NREM

## Abstract

Simultaneous electroencephalography and functional magnetic resonance imaging (EEG–fMRI) studies have revealed brain activations time-locked to spindles. Yet, the functional significance of these spindle-related brain activations is not understood. EEG studies have shown that inter-individual differences in the electrophysiological characteristics of spindles (e.g., density, amplitude, duration) are highly correlated with “Reasoning” abilities (i.e., “fluid intelligence”; problem solving skills, the ability to employ logic, identify complex patterns), but not short-term memory (STM) or verbal abilities. Spindle-dependent reactivation of brain areas recruited during new learning suggests night-to-night variations reflect offline memory processing. However, the functional significance of stable, trait-like inter-individual differences in brain activations recruited during spindle events is unknown. Using EEG–fMRI sleep recordings, we found that a subset of brain activations time-locked to spindles were specifically related to Reasoning abilities but were unrelated to STM or verbal abilities. Thus, suggesting that individuals with higher fluid intelligence have greater activation of brain regions recruited during spontaneous spindle events. This may serve as a first step to further understand the function of sleep spindles and the brain activity which supports the capacity for Reasoning.

## Introduction

Sleep spindles are one of the defining features of non-rapid eye movement (NREM) sleep. Spindles are traditionally defined as bursts of waxing and waning neural oscillations between 11 and 16 Hz (Iber et al., 2007), which stand out from the ongoing, background electroencephalographic (EEG) activity (Rechtschaffen and Kales, 1968). The brain regions activated during spontaneous spindle events have been identified (Laufs et al., 2007; Schabus et al., 2007; Tyvaert et al., 2008; Andrade et al., 2011; Caporro et al., 2012). However, the functional significance of these brain activations has yet to be elucidated, thereby limiting our understanding of the function of sleep spindles. Spindles are remarkably stable from night-to-night, but vary considerably from one individual to another, and because of the trait-like nature of spindles (Silverstein and Levy, 1976), they have even been suggested to be an “electrophysiological fingerprint” (De Gennaro et al., 2005). Recent work by our group and others (Nader and Smith, 2001, 2003; Bódizs et al., 2005, 2008; Schabus et al., 2006; Fogel et al., 2007; Ujma et al., 2014, 2015; Fang et al., 2017) suggests that spindles are electrophysiological markers of specific cognitive abilities, and in particular, Reasoning abilities (i.e., “fluid intelligence”; problem solving skills, the ability to employ logic, identify complex patterns). However, the neural basis of this relationship is not known; there is no direct evidence investigating the relationship between trait-like cognitive abilities and interindividual differences in spindle-dependent brain activations. Thus, as a first step, we sought to explore what brain activations time-locked to spontaneous spindle events are correlated with various domains of human intellectual abilities. This might shed light on the functional significance of brain activations time-locked to sleep spindles, and the neural functional correlates which explain individual differences in trait-like cognitive strengths and weaknesses.

In an effort to identify the functional brain areas recruited during spontaneous spindle events, a handful of studies have employed simultaneous electroencephalography and functional magnetic resonance imaging (EEG–fMRI) to explore brain activations time-locked to spindles (Laufs et al., 2007; Schabus et al., 2007; Tyvaert et al., 2008; Andrade et al., 2011; Caporro et al., 2012). Spindle-related activations have been consistently found in the thalamus and the temporal lobe, for both fast spindles and slow spindles (Laufs et al., 2007; Schabus et al., 2007; Tyvaert et al., 2008; Andrade et al., 2011; Caporro et al., 2012), as well as activation of the cingulate cortex and motor areas (Andrade et al., 2011; Caporro et al., 2012). Interestingly, activation of the putamen has also been found to be associated with spindle events (Tyvaert et al., 2008; Caporro et al., 2012) and Andrade et al. (2011) found a strong interaction between spindle occurrence and hippocampal formation functional connectivity, suggesting that spindles may be related to memory and cognitive functioning. However, they did not report any relationship between these activations and cognitive abilities. In addition, by directly comparing fast spindles vs. slow spindles, Schabus et al. (2007) observed increased activations associated with slow spindles in the superior temporal gyrus while fast spindles recruited activation in sensorimotor areas, mesial frontal cortex, hippocampus, and cerebellum; important memory centers of the brain. However, in the absence of any direct relationship between spindle-dependent activation and cognitive performance, the functional significance of these spindle-related activations could only be speculated based alone on the brain regions recruited. Taken together, the extant literature, not-surprisingly, suggests that brain activations associated with the action of sleep spindles involve well-known spindle-generating regions (e.g., thalamic and cortical regions), as well as, more intriguingly, regions which subserve executive functioning [prefrontal cortex (PFC)], declarative memory (hippocampus), motor skills (motor cortex and cerebellum), and procedural memory (the striatum). However, these studies are limited in that they can only tell us what brain regions are recruited during spindle events. The functional significance of these activations with respect to the trait-like nature of spindles remains to be elucidated.

Notably, the sleep spindle is the only known spontaneous neural oscillation that has been identified as an electrophysiological marker of cognitive abilities and aptitudes, that are typically assessed by intelligence quotient (IQ) tests (for review, see Fogel and Smith, 2011). The association between sleep spindles and individual differences in cognitive abilities has been well documented. More specifically, previous studies have revealed that interindividual differences in spindle characteristics are related to the capacity for Reasoning (i.e., the ability to identify complex patterns and relationships, the use of logic, existing knowledge, skills, and experience to solve novel problems (Nader and Smith, 2001, 2003; Bódizs et al., 2005, 2008; Schabus et al., 2006; Fogel et al., 2007; Ujma et al., 2014, 2015; Fang et al., 2017). For example, Nader and Smith (2001, 2003) found that both the number of sleep spindles and sigma power (12–14 Hz) was correlated with Performance IQ scores. Further studies revealed this relationship to be specific to these Reasoning-related abilities, over-and-above (i.e., controlling for) Verbal IQ (Fogel et al., 2007; Fang et al., 2017). Consistently, several studies (Bódizs et al., 2005; Schabus et al., 2006) found that fast spindles were positively correlated with similar metrics of Reasoning abilities (i.e., “fluid intelligence”), measured by the Raven’s Progressive Matrices (Raven et al., 1976). Similar studies identified a positive correlation between right-parietal fast spindles and visuospatial abilities assessed by the Rey–Osterrieth complex figure test (Bódizs et al., 2008). They identified a positive correlation between spindles and intellectual abilities measured by the Cattell Culture Fair Intelligence test, specifically in woman but not in men (Ujma et al., 2014). Although a relationship in men was subsequently identified by the same group in daytime sleep (Ujma et al., 2015). Taken together, these studies support the notion that sleep spindles are an electrophysiological marker of cognitive abilities, and specifically, the ability to solve problems using logic and Reasoning. These studies have provided insight into the electrophysiological correlates of Reasoning abilities, insofar as to indirectly suggest that efficient functioning of the neural substrates that support spindle generation and those that are recruited during spontaneous spindle events may be related to the capacity for these cognitive skills. Two recent studies employing simultaneous EEG–fMRI have investigated the association between sleep spindle-related brain activation and memory consolidation, including declarative (Bergmann et al., 2012) and procedural memory (Fogel et al., 2017a). However, both of these studies were interested in investigating memory trace reactivation following new learning (i.e., acute effects of learning on subsequent sleep), and did not investigate the stable, trait-like interindividual differences in cognitive abilities and how they relate to inter-individual differences in spindle-dependent brain activation. Thus, the functional significance of spontaneous spindle-related brain activations, and whether these activations related to specific domains of cognitive abilities [e.g., Reasoning, Verbal, Short-Term Memory (STM)] in healthy individuals remains to be investigated, which is the principle aim of the current study.

Therefore, here, using simultaneous EEG–fMRI recordings during sleep, we sought to identify, for the first time, the neuroanatomical function correlates of the well-established relationship between sleep spindles and specific cognitive abilities. We hypothesized that the neural activation patterns, time-locked to spindles would be related to distinct cognitive abilities whereby, consistent with previous cognitive and EEG studies, spindle-related brain activations would be correlated to a greater extent with Reasoning, but not STM or Verbal abilities, and include brain regions known to be involved in spindle generation, and also known to support Reasoning abilities (e.g., thalamus, PFC, striatum, cerebellum). This will provide insight into the functional significance of sleep spindles.

## Materials and Methods

### Participants

To be included in the study, all participants were non-shift workers and medication-free; had no history of head injury or seizures; had a normal body mass index (<25); and did not consume excessive caffeine, nicotine, or alcohol. Interested participants had to score <10 on the Beck Depression (Beck et al., 1974) (BDI) and the Beck Anxiety (Beck et al., 1988) (BAI) inventories and have no history or signs of sleep disorders, indicated by the Sleep Disorders Questionnaire (Douglass et al., 1994). Extreme morning and evening types were excluded based on the Morningness–Eveningness Questionnaire (Horne and Ostberg, 1976). A total of 35 healthy right-handed adults (20 female) between 20 and 35 years old (*M* = 23.69, *SD* = 3.57) who met these initial screening criteria were recruited to participate in this study. In addition, participants were given a letter of information, provided informed written consent before participation, and were financially compensated for their participation. All study procedures and methods adhered to the Declaration of Helsinki and were approved by the Western University Health Science research ethics board.

To ensure the absolute minimum amount of data required for EEG and fMRI analyses, and to ensure a minimum sleep duration, quality, and continuity of sleep, participants were required to sleep for a period of at least 5 min of uninterrupted NREM sleep during the sleep session in the MRI scanner to be included in the analyses. Among the 35 participants recruited in the study, only 5 participants did not meet the 5-min consolidated NREM sleep criteria for the sleep session, and one participant did not complete the Cambridge Brain Sciences (CBS) online test. Therefore, 29 participants (*M* = 23.97, *SD* = 3.83, 17 female) were included in the final data analyses, that slept on average for 44 min. The detailed sleep architecture and sleep spindle parameters are reported in the section “Results” and Table 1.

**Table 1:** Sleep architecture and sleep spindle parameters for spindles during NREM sleep from EEG–fMRI recording sessions.

	*M*	*SD*
	**Sleep architecture**	
	
Wake (min) (*N* = 26)	26.87	20.25
NREM1 (min) (*N* = 26)	5.84	4.38
NREM2 (min) (*N* = 29)	23.87	14.50
SWS (min) (*N* = 20)	14.77	17.17
NREM (min)	39.29	19.33
REM (*N* = 8)	17.80	10.76
Total sleep	44.20	23.84
Sleep latency	8.16	10.11
	**Total bandwidth (11–16 Hz) spindles at Cz**	
	
Number	334.74	212.29
Duration (s)	0.49	0.05
Amplitude (μV)	27.21	6.43
Density	8.22	2.34


*NREM, non-rapid eye movement sleep; NREM1, stage 1 sleep; NREM2, stage 2 sleep; SWS, slow wave sleep; REM, rapid eye movement sleep.*

The necessary sample size was determined *a priori* based on previous studies, and power calculated, where possible using G^∗^Power for Mac version 3.1 (Faul et al., 2007, 2009). Based on the most comparable simultaneous EEG–fMRI studies (Laufs et al., 2007; Schabus et al., 2007; Tyvaert et al., 2008; Andrade et al., 2011; Caporro et al., 2012), previous studies have employed sample sizes *N* < 15. A recent study by our group using the same cognitive tests as the current study (Fang et al., 2017) found robust associations between spindles and cognitive abilities in a sample size of *N* = 24, replicating previous findings in smaller samples (e.g., *N* < 12; Fogel and Smith, 2006; Fogel et al., 2007). Based on power calculation for correlation with *p* (two-tailed) = 0.05 (*b* = 0.20, effect size = 0.56) (Fang et al., 2017), an *N* = 22 was required. Thus, *N* = 29 subjects included in this study was considered to provide adequate statistical power for the main effects of interest.

### Cognitive Ability Test

The CBS platform is a web-based test battery^1^, which has previously been used in large-scale (Hampshire et al., 2012; Wild et al., 2018) and smaller-scale studies (Brewer-Deluce et al., 2017; Fang et al., 2017; Fogel et al., 2018; Phan et al., 2018). The CBS trials include 12 cognitive tests that measure a broad range of cognitive abilities including reasoning, problem solving, planning, attention, and memory. The CBS trials are advantageous as the 12 tasks are adapted from well-known, well-established paradigms from the cognitive neuroscience literature, that assess a wide range of aspects of cognition. As opposed to conventional tests which are based solely on the face-validity of the constructs of interest, CBS subscales are derived quantitatively from a data-driven approach using factor analysis, conducted on a large population from a previous study (Hampshire et al., 2012). In addition, the CBS test is non-verbal in nature and computerized. Therefore, it has the advantage of ease of administration, and is also not dependent on verbal comprehension. In addition, automated scoring and consistent test administration minimizes error. More importantly, and particularly for the aims of the current study, sleep spindles have been found to be correlated with Reasoning ability scores derived from the CBS test battery, using the same testing approach used here (Fang et al., 2017). In addition, the neural correlates of each factor have been investigated previously using neuroimaging (Hampshire et al., 2012). Therefore, we chose the CBS platform to investigate the neural correlates between sleep spindles and cognitive abilities.

All 12 subtests are based on classic paradigms from cognitive psychology. For example, the Reasoning factor is best described in terms of performance on five tests adapted from the cognitive literature, including deductive reasoning (Cattell, 1940), spatial rotation (Silverman et al., 2000), feature match (Treisman and Gelade, 1980), spatial planning (Shallice, 1982), and polygons (Folstein et al., 1975). STM is best described in terms of four tests, including visuospatial working memory (Inoue and Matsuzawa, 2007), spatial span (Corsi, 1972), paired associates (Gould et al., 2006), and self-ordered search (Collins et al., 1998). Finally, verbal ability is best captured by performance on three tests, including verbal reasoning (Baddeley, 1968), color-word remapping (Stroop, 1935), and digit span (Wechsler, 1981). More detailed information of the 12 subtests can be found in the Supplementary Material.

During the orientation session, participants were informed about the study requirements and procedures, and given detailed instructions of the online CBS tests. All participants were required to register and complete the CBS tests online at home after the orientation session. Participants were required to use a computer mouse to perform the task. Completion of the 12 tests takes between 30 and 60 min in total. The order of the tasks was randomized across participants. Prior to each test, specific instructions on how to perform the respective test were displayed onscreen.

#### CBS Scores Calculation

Consistent with the previous literature (Hampshire et al., 2012), the raw scores from each of the 12 subtests were normalized using the mean and standard deviation obtained from a large, young population (*N* = 44,600; age 20–35 years) of subjects who completed the CBS Trials (Hampshire et al., 2012) (Equation 1). Each subtest was then weighted according to the factor loadings from Hampshire et al. (2012) (Equation 2). Finally, the respective sub-tests were averaged to create the Reasoning, STM, and Verbal sub-scales and transformed to standard scores (Equation 3), so that test scores were readily comparable to results from similar studies that employed test batteries tapping into Reasoning and Verbal abilities, such as the Multidimensional Aptitude Battery – II (Fogel and Smith, 2006; Fogel et al., 2007) and other commonly used batteries of cognitive abilities (e.g., Wechsler Adult Intelligence Scale (Wechsler, 1981). The descriptive statistics of each subtest are shown in Table 2.

**Table 2:** Descriptive statistics of the three CBS subscales (Reasoning, STM, and Verbal abilities).

IQ measures	Range	Mean ± SD	Median
Reasoning	78.84–108.17	95.65 ± 7.20	96.46
STM	84.38–115.33	101.60 ± 6.77	102.30
Verbal	88.51–110.92	99.62 ± 5.12	99.52


(1) $$Z-score=(X_{raw}-M_{norm})/{SD}_{norm}$$

(2) Weighted Score = Z−score*factor loadings

(3) Standard score=100+Weighted Score* 15

*where X*_raw_, *raw score of each test item in the current study; M*_norm_, *mean score of each test item from the literature; SD*_norm_, *standard deviation of each test item from the literature; factor loading*, *weight of each test item from the literature.*

### Experimental Procedure

**FIGURE 1:**
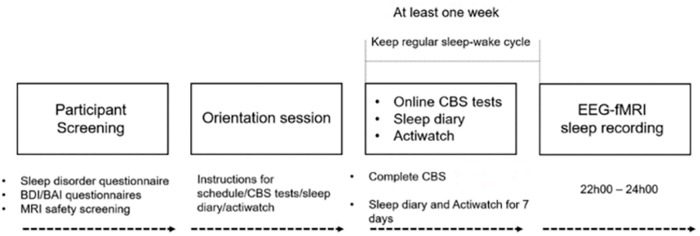
Experimental procedure of the study. Participants underwent initial screening to exclude any signs of sleep disorders, unusual sleep habits, or other health-related ineligibility and MRI compatibility. Eligible participants visited the sleep lab for the orientation session at least a week prior to the EEG–fMRI sleep recording night, in which participants were given detailed instructions about the study procedure, CBS tests, sleep diary, and actiwatch. All participants completed the CBS tests online and kept a regular sleep-wake cycle for at least 1 week prior to the sleep recording. Compliance with this schedule was assessed using both sleep diaries and wrist actigraphy. Participants who met these requirements were scheduled for the EEG–fMRI sleep recording session. MRI scanning started at 21h00, and lights out was from 22h00 to as late as 24h00.

An overview of the study procedure is shown in Figure 1. Participants underwent an initial screening prior to the study by completing the Sleep Disorder Questionnaires (Douglass et al., 1994), BDI (Beck et al., 1974), BAI (Beck et al., 1988) scales, Horne–Ostberg Morningness–Eveningness Questionnaire (Horne and Ostberg, 1976), and the MRI safety screening questionnaire to screen for signs of sleep disorders, unusual sleep habits, depression, or anxiety and MRI compatibility. Participants who met all these criteria were included in the study and visited the sleep lab for an orientation session at least 1 week prior to the EEG–fMRI sleep recording night. During the orientation session, participants were informed about the study requirements and procedures, and given detailed instructions of the online CBS tests. All participants were required to complete the CBS tests online at home after the orientation session, during which participants were not allowed to consume caffeinated, alcoholic, or nicotine products. Participants were required to keep a regular sleep-wake cycle (bed-time between 2200 and 2400 h, wake-time between 0700 and 0900 h), to abstain from taking daytime naps at least 7 days prior to and throughout participation in the study. Compliance with this schedule was assessed using both sleep diaries and wrist actigraphy (Actiwatch 2, Philips Respironics, Andover, MA, United States) worn on the non-dominant wrist for 1 week prior to the EEG–fMRI sleep recording night. Participants who met these requirements were scheduled for the EEG–fMRI sleep recording session. The experimental sleep session started between 21h00 and 24h00, during which time simultaneous EEG–fMRI was recorded while participants slept in the scanner. Specifically, the scan procedure normally started at 21h00, at which point, the EEG equipment was installed and tested. This was followed by localizer scans, a T1 MPRAGE structural scan, and an awake resting scan. These procedures took more than 30 min to complete. The sleep recording (“lights out”) normally started after 22h00, within the range of the subject’s habitual bedtime. The average sleep latency was 8.16 ± 10.11 min (Table 1) and the average sleep onset time, when participants fell asleep in the scanner was 22h22 (±25 min). Following the EEG–fMRI sleep session, participants were allowed to sleep in the nearby sleep laboratory for the remainder of the night.

### Polysomnographic Recording and Analysis

#### Recording Parameters

Simultaneous polysomnography was comprised of 64-channel MR-compatible EEG cap which included one electrocardiogram (ECG) lead (Braincap MR, Easycap, Herrsching, Germany) and two MR-compatible 32-channel amplifiers (Brainamp MR plus, Brain Products GmbH, Gilching, Germany). EEG recordings were taken referenced to FCz. Skin resistance was reduced below 5 KOhm using high-chloride abrasive electrode paste (Abralyt 2000 HiCL; Easycap, Herrsching, Germany). The single drop-down ECG electrode from the EEG cap can have less than optimal visualization of the R-peak of the QRS complex. For this reason, we used additional bipolar electrodes for three ECG derivations to obtain high-quality recordings in order to accurately identify *R*-peaks, using an MR-compatible 16-channel bipolar amplifier (Brainamp ExG MR, Brain Products GmbH, Gilching, Germany). This was done in order to increase the chances of acquiring at least one ECG channel with high quality *R*-peaks, necessary for effective ballistocardiographic (BCG) correction. In addition, as recommended by Mullinger et al. (2011), we repositioned subjects in the MRI scanner so that the subjects were shifted away from iso-center of the magnetic field by 40 mm. At this position, the MRI images are not impacted, but the BCG artifact has been reported to be reduced by up to 40%, thereby improving EEG quality after BCG correction. EEG data were transferred via fiber optic cables to a personal laptop where Brain Products Recorder Software, Version 1.x (Brain Products, Gilching, Germany) was synchronized to the scanner clock. Data were digitized with a resolution of 500-nv/bit at 5 kHZ and were analog filtered by a band-limiter low pass filter at 500 Hz and a high pass filter with a 10-s time constant corresponding to a high pass frequency of 0.0159 Hz.

#### EEG Data Processing

Electroencephalographic data were first corrected for gradient-induced and cardioballistic artifacts in two separate steps: In the first step, MRI gradient artifacts were removed using an adaptive average template subtraction method (Allen et al., 2000) implemented in Brain Products Analyzer, and down-sampled to 250 Hz. Sleep-fMRI parameters were chosen to ensure that the lowest residual gradient artifacts (18.52 Hz) would not compromise the sleep spindle frequency (11–16 Hz). Due to safety and data quality concerns, the Helium pump was not allowed to be switched off during the EEG–fMRI acquisition. However, through the pilot tests, we confirmed that the frequency of the pump noise is >80 Hz, which is outside EEG frequencies of interest in our study (e.g., 11–16 Hz for spindles), and would not impact slower frequencies needed for accurate visual sleep scoring. In the second step, the *R*-peaks in the ECG were semi-automatically detected, visually verified, manually adjusted when necessary, to correct both false positives and false negative *R*-peak detections. Then, adaptive template subtraction (Allen et al., 1998) was used to remove BCG artifacts time-locked to the *R*-peak of the QRS complex of the cardiac rhythm. After these two steps, we visually verified the quality of the data and inspected the amplitude of the residual artifacts time-locked to the *R*-peaks (Supplementary Figure S1). An independent component analyses (ICAs)-based approach (Srivastava et al., 2005; Mantini et al., 2007) was applied to remove any remaining BCG residual artifact if the peak of the maximum amplitude of the residual artifact exceeded 3 μV during the QRS complex (e.g., 0–600 ms). Finally, a low-pass filter (60 Hz) was applied to the EEG data, which were then re-referenced to averaged mastoids. A sample of the EEG traces after correction is shown in Supplementary Figure S2.

Following the artifact correction, sleep stages were scored in accordance with standard criteria (Iber et al., 2007) using the “VisEd Marks” toolbox^2^ for eeglab (Delorme and Makeig, 2004). Automatic spindle detection was carried out using a previously published and validated (Ray et al., 2015) method (*n.b.*, against both multiple expert scorers and using crowd-sourcing from a large sample of non-experts) employing EEGlab-compatible (Delorme and Makeig, 2004) software^3^ written for MATLAB R2014a (The MathWorks Inc., Natick, MA, United States). The detailed processing steps and procedures and validation are reported elsewhere (Ray et al., 2015) and are thus presented only briefly here. The spindle data were extracted from movement artifact-free, NREM sleep epochs. The detection method (Ray et al., 2015) used a complex demodulation transformation of the EEG signal with a bandwidth of 5 Hz centered about a carrier frequency of 13.5 Hz (i.e., 11–16 Hz) (Iber et al., 2007). Spindle detection was visually verified by an expert following automated detection. The variables of interest extracted from this method include spindle amplitude, duration, and density (number of spindles per minute of NREM sleep) for each participant and at each derivation (Fz, Cz, and Pz). However, given the limited amount of sleep in the current study, there was an insufficient number of spindle events to further subdivide spindles into slow (e.g., 11–13.5 Hz at Fz) and fast (e.g., 13.6–16 Hz at Pz) spindle types without excluding subjects due to missing data or insufficient number of spindle onsets. Thus, only results from full bandwidth spindles at Cz in NREM sleep were reported and included in the final analyses.

### Relationship Between Sleep Spindle EEG Characteristics and Cognitive Abilities

Linear regression analyses were used to examine the effects of sleep spindles on cognitive abilities (Reasoning, STM, and Verbal) assessed by the CBS test battery. Sleep spindle duration, amplitude, and density were entered into each model as dependent variables separately; Reasoning, STM, and Verbal subscale factors were entered into the models as independent variables. Inspection of the resulting partial correlation coefficients was conducted to identify which subscale (e.g., Reasoning, Verbal, or STM) accounted for the greatest proportion of unique interindividual variability for each spindle characteristic. The regression models included gender and whole brain volume as covariates of non-interest. These were included given that the relationship between spindles and Reasoning ability was reportedly different in men and women (Ujma et al., 2014), and brain volume might influence NREM slow wave oscillations (Saletin et al., 2013), sleep quality (Branger et al., 2016), or be related to cognitive abilities (Casey et al., 2005).

### MRI Imaging Acquisition and Analysis

#### Recording Parameters

Functional magnetic resonance imaging was performed at a 3.0T Magnetom Prisma MR imaging system (Siemens, Erlangen, Germany) using a 64-channel head coil. High-resolution anatomic images were acquired using a standard 3D Multislice MPRAGE sequence (TR = 2300 ms, TE = 2.98 ms, TI = 900 ms, FA = 9°, 176 slices, FoV = 256 × 256 mm^2^, matrix size = 256 × 256 × 176, voxel size = 1 × 1 × 1 mm^3^). During the sleep session, T2^∗^-weighted fMRI images were acquired with a gradient echo-planar imaging (EPI) sequence using axial slice orientation (TR = 2160 ms, TE = 30 ms, FA = 90°, 40 transverse slices, 3 mm slice thickness, 10% inter-slice gap, FoV = 220 × 220 mm^2^, matrix size = 64 × 64 × 40, voxel size = 3.44 × 3.44 × 3 mm^3^). Sleep-fMRI parameters were chosen to ensure that the lowest residual gradient artifacts (18.52 Hz) would not compromise the sleep spindle frequency (11–16 Hz). This was achieved by setting the MR scan repetition time to 2160 ms, such that it matched a common multiple of the EEG sample time (0.2 ms), the product of the scanner clock precision (0.1 μs), and the number of slices (40 slices) used (Mulert and Lemieux, 2009). Among all participants, up to 2 h of sleep EEG–fMRI data was acquired. An expert, registered polysomnographic technologist scored the EEG data acquired during the simultaneous EEG–fMRI sleep recordings according to standard criteria (Iber et al., 2007).

#### Image Preprocessing

Functional images were preprocessed and analyzed using SPM8^4^ (Welcome Department of Imaging Neuroscience, London, United Kingdom) implemented in MATLAB (ver. 8.5 R2015a) for Windows (Microsoft, Inc. Redmond, WA, United States). For each subject, functional images were corrected for slice acquisition time differences and realigned to correct head motion using rigid body transformation. A mean realigned image was then created from the resulting images. The structural T1-image was coregistered to this mean volume of functional images. Using DARTEL in SPM8, the coregistered structural images were segmented into gray matter, white matter, and cerebrospinal fluid, and an average subject-based template was created. All functional and anatomical images were then spatially normalized using the resulting template, which was generated from the structural scans. Finally, spatial smoothing was applied on all functional images (Gaussian kernel, 8 mm full-width at half-maximum (FWHM).

#### First-Level Individual GLM

The onset time and duration of each spindle were identified from the EEG data. Given that the EEG and fMRI recordings were recorded simultaneously and precisely synchronized, the brain activations time-locked to each spindle could be estimated using the onset of each spindle (converted to TR) and duration of each spindle in a fixed effects GLM using an even-related fMRI design. The BOLD time series data were modeled using a canonical hemodynamic response function (HRF). In total, 27 nuisance variables were entered in the model to be removed, including the Friston-24 movement parameters (Friston et al., 1996), the mean white matter intensity, and the mean cerebral spinal fluid intensity for each participant. In addition, the spectral power (μV^2^) in the delta band (0.5–4 Hz) for each TR window (2160 ms) was also entered as a nuisance variable, given that slow wave activity is a defining characteristic of NREM sleep (Iber et al., 2007), and is related to spindle generation (Siapas and Wilson, 1998; Mölle et al., 2011). High-pass filtering was implemented in the design using a cut-off at 128 s to remove low frequency drifts from the time series. These analyses generated spindle-related contrast maps of the *t*-statistic [SPM(*t*)] for all spindle events.

#### Second-Level Group GLM

The SPM(*t*) maps generated from the first-level GLM were entered into second-level GLMs for group-level analyses. One sample *t*-tests were conducted for the brain activations time-locked to spindles. In addition, to investigate the relationship between the magnitude of the spindle-dependent activations and the cognitive abilities assessed by the CBS subscales, whole-brain spatial multiple regression analyses were conducted at the group level using the SPM8 toolbox. Spindle-related contrast maps of the *t*-statistic [SPM(*t*)] from first-level GLM were selected, and cognitive test scores for each subtest (e.g., Reasoning, Verbal, and STM) were entered as covariates of interest in the described GLMs, separately. Gender and whole brain volume were included in the models as variables of non-interest. Multiple regression analyses were conducted at the whole-brain level voxel by voxel [i.e., no region of interest (ROI) mask was applied] to explore which brain regions recruited during spindle events were correlated to each CBS subtest. Statistical inferences were performed at a threshold of *p* < 0.001 (uncorrected) at the whole-brail level and *p* < 0.05, family wise error (FWE) corrected at the cluster level.

#### Statistical Comparisons of Brain–Behavior Correlations Among Three Cambridge Brain Sciences (CBS) Subtests

Reasoning ability was highly inter-correlated with Verbal ability (*r* = 0.596; *p* = 0.001), and marginally correlated with STM ability (*r* = 0.357; *p* = 0.058). To further test whether the relationship between Reasoning ability and the spindle-related activations could be accounted for by Verbal or STM abilities, we directly compared the correlations between spindle-related activations and three CBS subtests (Reasoning, Verbal, and STM). Due to high multicollinearity, we could not include all subtests scores into one design matrix model in SPM. Therefore, we employed a ROI approach for this comparison. We selected three main regions for further analyses, including the thalamus, the anterior cingulate cortex/middle cingulate cortex (ACC/MCC), and the bilateral putamen, because in the current study, we found the brain activation in these three regions were time-locked to spindle events and also correlated with Reasoning abilities. In addition, these three regions have been consistently reported to be recruited during spindle events in the extant literature (Schabus et al., 2007; Tyvaert et al., 2008). In order to employ an independent ROI analysis to avoid circularity (Kriegeskorte et al., 2009), the three ROIs were defined based on the coordinates reported in the previous literature which also used EEG–fMRI approach to investigate the spindle-related brain activation, using the MarsBaR toolbox^5^. The thalamus ROI was derived from Tyvaert et al. (2008) (L: -14, -10, 6; R: 19, -10, 5, radiums: 6 mm); the ACC/MCC was derived from Schabus et al. (2007) (ACC, L: -6, 34, 14; R: 6, 38, 12; MCC, -2, 26, 24, radiums: 6 mm); the bilateral putamen was derived from Tyvaert et al. (2008) (L: -29, -3, 7; R: 28, -7, 13, radiums: 6 mm). Using the MarsBaR toolbox, we calculated parametric estimate activation values in response to spindle events in the three independent ROIs and conducted standard multiple linear regressions. Brain activation parametric estimate values in the thalamus, the ACC/MCC, and the bilateral putamen were included in the models as dependent variables and Reasoning, Verbal, and STM scores were entered as independent variables. Gender and whole brain volume were included as covariates of non-interest.

#### Overlap Between Spindle-Related Maps and Reasoning-Spindle Correlation Maps

To illustrate the overlap of activations between the spindle-related activation maps and Reasoning-spindle correlation maps, the conjunction was taken as the minimum *t*-statistic using the conjunction null hypothesis (Friston et al., 2005; Nichols et al., 2005) over:

(1) a *t*-map testing for the main effect of the spindle events during the sleep session and
(2) a *t*-map testing for the main effect of the correlation between the Reasoning ability and spindle events.

These two statistical maps were thresholded at *p* < 0.001 (uncorrected) at the whole-brain level and *p* < 0.05, FWE corrected at the cluster level.

## Results

### Sleep Architecture

An overview of the study procedure is shown in Figure 1. Of the *N* = 35 participants recruited in the study, only 5 participants did not meet the 5-min consolidated NREM sleep criteria for the sleep session, and one participant did not complete the CBS online test. Therefore, *N* = 29 participants were included in the final analyses. As shown in Table 1, among these 29 participants, *N* = 26 participants experienced NREM1 sleep, all *N* = 29 participants experienced NREM2 sleep, *N* = 20 had SWS sleep, and *N* = 8 had rapid eye movement (REM) sleep. The (*N* = 29) participants had at least 14.67 min of sleep, and on average, a total of 44.20 (*SD* = 23.84) min of sleep (including REM) during the sleep recording session in the MRI scanner. The average sleep latency of these 29 participants was 8.16 ± 10.11 min and the average sleep onset time when participants fell asleep in the scanner was 22h22 (±25 min). Given the focus of the current investigation on NREM spindles, we analyzed only the NREM data of the 29 subjects. The average duration of NREM sleep was 39.29 (*SD* = 19.33) min and participants had on average 334.74 (*SD* = 212.29, min = 63, max = 1035) sleep spindles during NREM sleep.

### Relationship Between Sleep Spindle Characteristics and Cognitive Abilities

**FIGURE 2:**
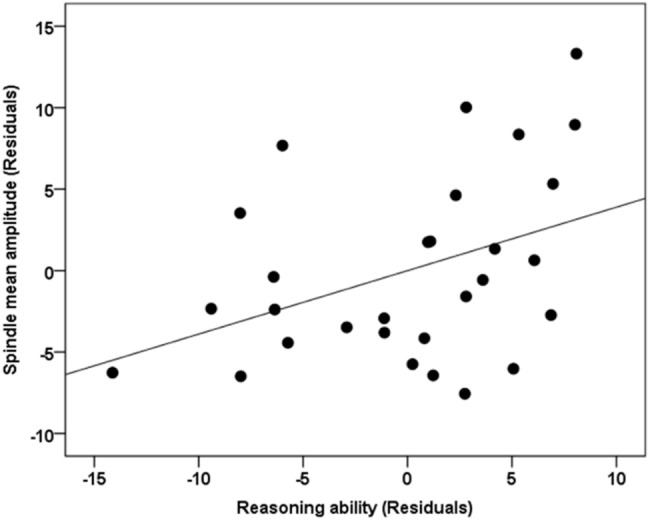
Correlation between spindle amplitude and Reasoning ability. The unique relationship (i.e., partial correlation, *r* = 0.435, *p* = 0.030) between Reasoning ability, controlling for STM and Verbal abilities with spindle amplitude during NREM sleep. Gender and the whole brain volume were included in the model as covariates of no interest. Values reported in the scatterplot are residuals (showing the partial correlation) and shown in standardized arbitrary units.

Standard multiple linear regression analyses revealed that, taken together, Reasoning, STM, and Verbal abilities assessed by the CBS trials (Table 2) significantly accounted for variability in spindle amplitude [*F*(5,23) = 3.424, *R*^2^ = 0.427, *p* = 0.019], but not duration [*F*(5,23) = 0.366, *R*^2^ = 0.074, *p* = 0.867), or density [*F*(5,23) = 1.489, *R*^2^ = 0.245, *p* = 0.232] during NREM sleep (Table 3). Similar to previous studies (Fogel et al., 2007; Fang et al., 2017), follow-up inspection of the partial coefficients revealed that Reasoning ability, controlling for STM and Verbal abilities [*t*(23) = 2.314, *r* = 0.435, *p* = 0.030] accounted for variability in spindle amplitude (Figure 2). By contrast, neither STM, controlling for Reasoning and Verbal abilities [*t*(23) = 0.394, *r* = 0.082, *p* = 0.697), nor Verbal abilities, controlling for STM and Reasoning abilities [*t*(23) = 0.642, *r* = 0.133, *p* = 0.527) accounted for variability in sleep spindle amplitude. Thus, Reasoning abilities (but not STM or Verbal abilities) were uniquely related to spindle characteristics. To further explore which subtest score (i.e., deductive reasoning, spatial rotation, feature match, spatial planning, and polygons) from the Reasoning subscale was correlated with spindle amplitude, partial correlation analysis revealed that deductive reasoning, spatial planning, and polygons were all significantly correlated with spindle amplitude (*p* < 0.05), while spatial rotation and spatial planning were marginally correlated (*p* < 0.10) with spindle amplitude (Supplementary Table S1).

**Table 3:** Multiple regression analyses of the relationship between CBS trials and spindles (Figure 2).

	Overall regression effect		
	
Sleep spindle parameter	Unadjusted *R*^2^	*F*(5,23)	*p*
Amplitude	0.427	3.424	0.019^*^
Duration	0.074	0.366	0.867
Density	0.245	1.489	0.232

	**Follow-up analyses for amplitude**		
	
**CBS measures**	**Semipartial *r***	***t*(23)**	***p***

Reasoning	0.435	2.314	0.030^*^
Verbal	0.133	0.642	0.527
STM	0.082	0.394	0.697


*Statistically significant results indicated by an asterisk (^∗^) at *p* < 0.05; STM, short-term memory.*

### Activation of Brain Regions Time-Locked to Spindles During NREM Sleep

**FIGURE 3:**
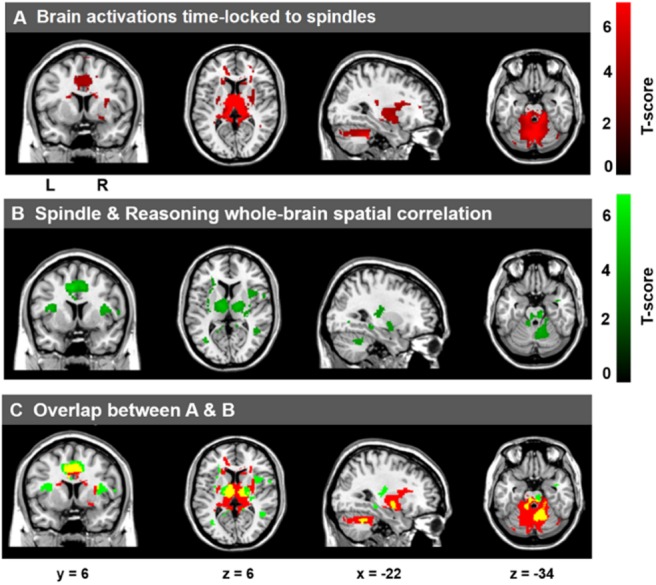
Cerebral activations time-locked to sleep spindles and correlation between spindle-related activation and Reasoning abilities. **(A)** Activations time-locked to sleep spindles during NREM sleep. **(B)** Spatial correlation maps between activations time-locked to sleep spindles and Reasoning abilities. **(C)** Overlap between **A** (red) and **B** (green), with the conjunction of **A** and **B** shown in yellow. Statistical inferences were performed at a threshold of *p* < 0.001 (uncorrected) at the whole-brail level and *p* < 0.05, FWE corrected at the cluster level.

As expected, and consistent with previous studies (Laufs et al., 2007; Schabus et al., 2007; Tyvaert et al., 2008; Andrade et al., 2011; Caporro et al., 2012), as shown in Figure 3A, activations time-locked to spindles were observed in the thalamus/midbrain, the bilateral striatum (putamen/globus pallidus and caudate), the medial frontal cortex, the cerebellum, and the brain stem (statistical inferences were performed at a threshold of *p* < 0.001 uncorrected at the whole-brain level and *p* < 0.05, FWE corrected at the cluster level, Table 4).

**Table 4:** Statistically significant activations time-locked to sleep spindles (Figure 3A).

Hemisphere	Region	MNI coordinate			Peak *z-*score	FWE-corrected *p*-value
				
		*X*	*Y*	*Z*		
Right	Thalamus	10	-22	10	6.14	<0.001
Left	Thalamus	-12	-24	18	6.03	<0.001
Left	Caudate	-14	12	12	6.07	<0.001
Left	Putamen/pallidum	-18	-2	-4	4.22	0.001
Right	Putamen/pallidum	18	-4	-4	5.32	<0.05
Bilateral	Cerebellum	2	-62	-10	5.88	<0.001
Left	Anterior cingulate	-16	32	18	5.16	0.001
Right	Anterior cingulate	12	22	28	3.94	0.001
Middle	Middle cingulate	0	24	32	4.53	0.001


*Significant brain responses after FWE correction *p* < 0.05 at the cluster level.*

### Correlation Between Brain Activations Time-Locked to Spindles and Cognitive Abilities

**FIGURE 4:**
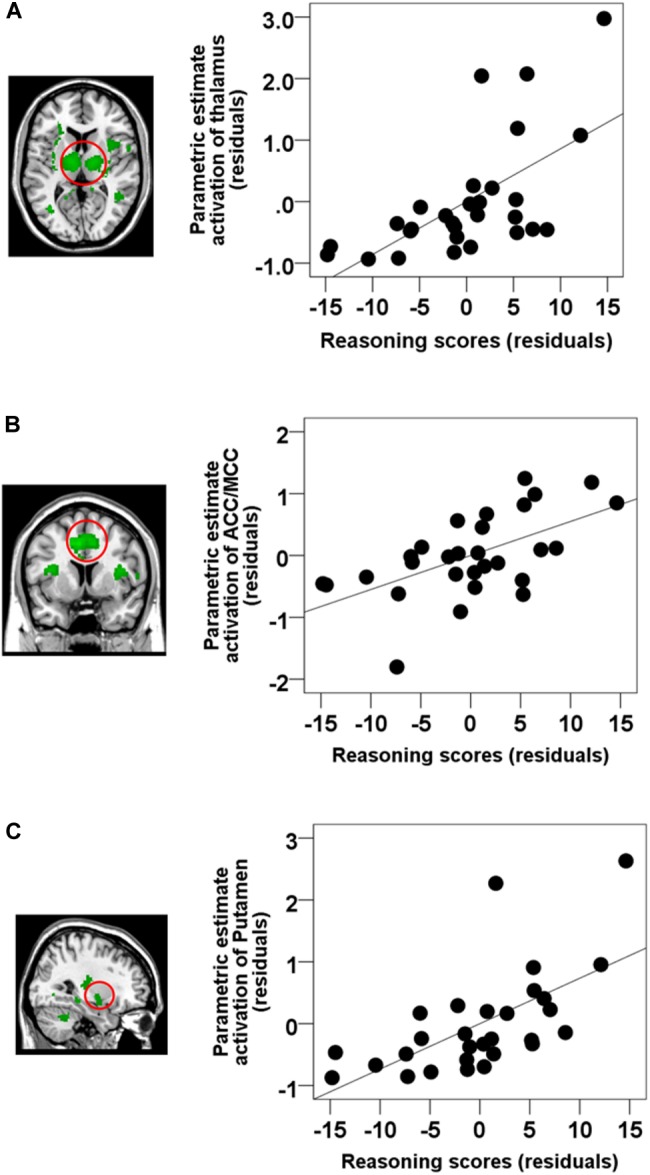
Semi-partial correlations between brain activations time-locked to spindles and Reasoning scores. ROI analyses revealed that Reasoning abilities were correlated with activations in the **(A)** the thalamus (partial correlation, *r* = 0.628, *p* < 0.001), **(B)** ACC/MCC (partial correlation, *r* = 0.585, *p* = 0.001), and **(C)** the bilateral putamen (partial correlation, *r* = 0.616, *p* = 0.001). Gender and the whole brain volume were included in the model as covariates of no interests. Values are standardized residuals (showing the partial correlation) and shown in standardized arbitrary units.

To examine the correlation between the level of activation of neural substrates recruited during spindles events and cognitive abilities, we conducted whole-brain spatial correlation analyses between brain activation maps time-locked to spindles and the scores on the three distinct cognitive subscales (Reasoning, STM, and Verbal abilities) assessed by the CBS trials. As shown in Figure 3B, Reasoning ability was significantly correlated with activations time-locked to spindle events in the thalamus, bilateral putamen, brainstem/pons, ACC, the MCC, the paracentral lobe, the posterior cingulate cortex, the precuneus, and bilateral temporal lobe (statistical inferences were performed at a threshold of *p* < 0.001 uncorrected at the whole-brail level and *p* < 0.05, FWE corrected at the cluster level (Table 5). To illustrate these relationships more clearly, the semi-partial correlations are shown in Figure 4. The ROIs of ACC/MCC, thalamus, and bilateral putamen were defined independently based on the previous literature, as described in the “Materials and Methods” section.

**Table 5:** Whole brain correlations between Reasoning ability and spindle-related activations (Figure 3B).

Hemisphere	Region	MNI coordinate			Peak *z-*score	Cluster-level FWE corrected *p*-value
				
		*X*	*Y*	*Z*		
Left	Paracentral lobule	-12	-32	58	4.83	<0.001
Middle	Anterior cingulate	-6	12	26	4.19	<0.001
Middle	Middle cingulate	-6	10	42	4.30	<0.001
Left	Precuneus	-14	-58	32	4.83	<0.001
Left	Putamen/pallidum	-16	-6	-2	4.34	<0.001
Left	Thalamus	-12	-10	6	3.99	<0.001
Right	Thalamus	16	-10	8	3.88	<0.001
Right	Brain stem	14	-32	-32	4.58	<0.001
Left	Brain stem	-8	-30	-26	4.09	<0.001
Right	Cerebellum	14	-64	-34	3.94	<0.001
Left	Temporal lobe	-42	-58	-2	4.01	<0.05
Right	Temporal lobe	48	-52	-4	4.14	<0.005


*Significant brain responses after FWE correction *p* < 0.05 at the cluster level.*

Remarkably, no activations time-locked to spindle events were significantly related to STM or Verbal ability when considered as the covariate of interest (cluster-level *FWE* correction, *p* < 0.05). Moreover, very few results were significant even at a much less conservative, *p* < 0.001, without correction for multiple comparisons for Verbal abilities, and no results for STM at that threshold.

### Comparisons of Brain–Behavior Correlations Between Reasoning, STM, and Verbal Abilities

Reasoning ability was highly inter-correlated with Verbal ability (*r* = 0.596, *p* = 0.001), and marginally correlated with STM ability (*r* = 0.357, *p* = 0.058), thus preventing all three scales to be included in a whole brain regression analysis due to high multicollinearity. Thus, in order to further examine whether the significant relationship between activations time-locked to spindle events and Reasoning ability could be accounted for by STM or Verbal abilities, we selected three ROI based on the spindle activations consistently observed in the extant literature (see the section “Materials and Methods” for details). ROIs included: the thalamus, the ACC/MCC, and the bilateral putamen. We extracted the beta weights from these three independent ROIs from coordinates determined from the literature. We then conducted standard multiple linear regressions whereby parametric brain activation estimate values in the thalamus, the ACC/MCC, and the bilateral putamen were included in the models as dependent variables, and Reasoning, Verbal, and STM scores were included in the models as independent variables. Similar to the whole-brain analysis (Table 6), altogether, Reasoning, STM, and Verbal abilities significantly accounted for brain activation time-locked to spindles in the thalamus (*R*^2^ = 0.425, *p* = 0.019), the cingulate cortex (*R*^2^ = 0.627, *p* = 0.033), and the putamen (*R*^2^ = 0.456, *p* = 0.011). Importantly, follow-up inspection of the partial coefficients revealed that Reasoning ability uniquely accounted for spindle-related activation in the thalamus (*r* = 0.460, *p* = 0.021), the cingulate cortex (*r* = 0.434, *p* = 0.030), and the putamen (*r* = 0.458, *p* = 0.021) over and above Verbal and STM (i.e., controlling for STM and Verbal abilities). The same partial correlation analyses were conducted between the five subtests scores and the spindle-related brain activation in the three main regions of interest (i.e., thalamus, ACC/MCC, putamen). As shown in Supplementary Table S2, all subtests were correlated with brain activations time-locked to spindles in thalamus and putamen (*p* < 0.05) except for feature match. In addition, the ACC/MCC activation was significantly correlated with deductive reasoning, spatial rotation, and polygons (*p* < 0.05), and marginally correlated with spatial planning (*p* = 0.06).

**Table 6:** Multiple regression analyses of the relationship between CBS trials and brain activation time-locked to spindles.

	Overall regression effect		
	
Correlated region	Unadjusted *R*^2^	*F*(5,23)	*p*
Thalamus	0.425	3.394	0.019^*^
ACC/MCC	0.627	2.974	0.033^*^
Putamen	0.456	3.855	0.011^*^
	**Follow-up analyses for the thalamus**		
	
**CBS measures**	**Partial *r***	***t*(23)**	***p***

Reasoning	0.460	2.486	0.021^*^
Verbal	0.210	1.029	0.314
STM	-0.091	-0.436	0.667
	**Follow-up analyses for the ACC/MCC**		
	
**CBS measures**	**Partial *r***	***t*(23)**	***p***

Reasoning	0.434	2.307	0.030^*^
Verbal	0.229	1.128	0.271
STM	-0.198	-0.971	0.342
	**Follow-up analyses for the putamen**		
	
**CBS measures**	**Partial *r***	***t*(23)**	***p***

Reasoning	0.458	2.473	0.021^*^
Verbal	0.293	1.470	0.155
STM	-0.241	-1.193	0.245


*Statistically significant results indicated by an asterisk (^∗^) at *p* < 0.05; STM, short-term memory.*

### Overlap Between Activations Time-Locked to Spindles and Reasoning-Related Spindle Activations

From Figure 3, we can see that there were several overlapping regions between the spindle activation maps (Figure 3A) and the maps that show activations time-locked to spindles that were correlated with Reasoning abilities (Figure 3B). To follow-up this observation, the overlap (*p* < 0.05, FWE at the cluster level) between the spindle activation maps and the Reasoning-related spindle correlation maps (Figure 3C, yellow regions) show several regions were consistently high and jointly activated in both the spindle maps (Figure 3A) and Reasoning-spindle correlation maps (Figure 3B), including the thalamus, medial frontal cortex, bilateral putamen, and the cerebellum. Thus, suggesting that a subset of the spontaneous spindle-related activations was uniquely correlated with Reasoning abilities.

## Discussion

Advancements in simultaneous EEG–fMRI technology and techniques has enabled the investigation of the functional brain activation recruited during well-known electrophysiological events such as sleep spindles. This can provide a window into understanding what brain areas are involved in sleep processes and their function. Given the challenging nature of applying these techniques to study sleep, only a handful of studies have explored the brain activations correlated with sleep spindles (Laufs et al., 2007; Schabus et al., 2007; Tyvaert et al., 2008; Andrade et al., 2011; Caporro et al., 2012). While important in terms of advancing the understanding of the neurophysiology and functional anatomy of the spindle, unfortunately, we can only infer the functional significance of these brain activations from these studies. Thus, limiting our understanding of the related functions of sleep spindles and their neural correlates. Previous EEG and behavioral studies have identified sleep spindles as a biological marker of cognitive abilities, and in particular, Reasoning abilities (Bódizs et al., 2005; Schabus et al., 2006; Fogel et al., 2007; Fogel and Smith, 2011; Ujma et al., 2014, 2015; Fang et al., 2017). While providing important information about the functional significance of the sleep spindle, the neuroanatomical substrates and neural mechanisms which support the relationship between spindles and Reasoning abilities can only be inferred indirectly from these studies. Here, we identified the neural activation patterns time-locked to spindles that are correlated to cognitive abilities. Using simultaneous EEG–fMRI sleep recordings, the results of the present study support three main findings:

(1) similar to previous studies (Fogel et al., 2007; Fang et al., 2017), the electrophysiological spindle characteristics (e.g., amplitude) during NREM sleep were related to Reasoning but not STM or Verbal abilities,
(2) similar to previous studies (Laufs et al., 2007; Schabus et al., 2007; Tyvaert et al., 2008; Andrade et al., 2011; Bergmann et al., 2012; Caporro et al., 2012; Fogel et al., 2017a), activations time-locked to spindles were observed in the thalamus, bilateral striatum, MCC, and cerebellum, and importantly,
(3) Reasoning abilities, but not STM or Verbal abilities, were correlated with spindle-related activations in a subset of these regions including the thalamus, bilateral putamen, medial frontal gyrus, MCC, and precuneus.

These results provide evidence that individuals with greater neural activation time-locked to spindle events have greater Reasoning abilities (i.e., “fluid intelligence”; problem solving skills, the ability to employ logic, identify complex patterns). Altogether, our results identified for the first time, that a subset of spontaneous spindle-related activations are correlated specifically with Reasoning abilities but are unrelated to other abilities such as STM and Verbal abilities. Thus, suggesting that the extent of spindle-related activations reflect an individual’s capacity for reasoning.

### Association Between Spindle Amplitude and Reasoning Abilities

The three subtests (i.e., Reasoning, STM, and Verbal) that assess sub-domains of general cognitive abilities are highly inter-correlated with one another. However, among these three inter-correlated subtests, only Reasoning abilities were found to be correlated with spindles, when the overlapping variability was controlled using multiple regression. This finding has been reported previously whereby the electrophysiological characteristics of spindles were correlated with Reasoning abilities (Fang et al., 2017) and Performance intelligence (Fogel et al., 2007), but not Verbal abilities. Also, it is consistent with the extant literature and the well-established finding that spindles are related to a subset of cognitive abilities that tap into reasoning and the ability to solve problems, i.e., fluid intelligence (Nader and Smith, 2001, 2003; Schabus et al., 2006; Ujma et al., 2014, 2015). Using CBS tests, Hampshire et al. (2012) identified that the network of brain regions which support these three subtests of cognitive abilities were distinct from each other. Therefore, indicating that although these three subtest are inter-correlated with each other, they are associated with distinct neural substrates. Our findings further suggest that BOLD brain activations during spindle events are uniquely related to Reasoning abilities, over and above STM and Verbal abilities. It is also worth noting that according to previous studies (Bódizs et al., 2005; Schabus et al., 2006; Fogel et al., 2007; Fogel and Smith, 2011; Hoedlmoser et al., 2014; Róbert Bódizs et al., 2014; Ujma et al., 2014, 2015, 2016; Tessier et al., 2015; Fang et al., 2017), there is no single or unique spindle electrophysiological characteristic that is correlated with cognitive abilities (i.e., fluid intelligence, reasoning, problem solving, etc.). Several spindle parameters have been found to be correlated with cognitive abilities. For example, spindle amplitude (Bódizs et al., 2014; Ujma et al., 2014, 2016; Fang et al., 2017), spindle activity (duration × amplitude) (Schabus et al., 2006; Hoedlmoser et al., 2014; Tessier et al., 2015), and spindle density (Bódizs et al., 2005; Fogel et al., 2007) have all been reported to be correlated with cognitive abilities. In the current study, we observed the strongest association between spindle amplitude and reasoning ability. This is consistent with the finding of a recent meta-analysis study (Ujma, 2018), which reported that only spindle amplitude is unambiguously associated with cognitive ability.

### Spontaneous Brain Responses Time-Locked to Sleep Spindles

As expected, and consistent with previous EEG–fMRI studies of spontaneous spindle-related activations (Laufs et al., 2007; Schabus et al., 2007; Tyvaert et al., 2008; Andrade et al., 2011; Bergmann et al., 2012; Caporro et al., 2012; Fogel et al., 2017a), our results identified and confirmed the brain regions associated with spindle events during NREM sleep in both cortical (including the media prefrontal, ACC, and MCC), subcortical areas (including the thalamus and bilateral caudate, putamen, and pallidum), and the cerebellum. Thus, confirming the recruitment of cortico-thalamic-striatal circuitry in spindle generation. These human neuroimaging findings are supported by a large body of animal studies, which at the cellular level, suggest that spindles reflect oscillatory activity in widespread thalamocortical circuits, and involve complex interactions between reticular, thalamocortical, and pyramidal cells (Steriade, 2005). Unfortunately, due to the limited duration, and high inter-subject variability of sleep in the scanner, we did not have sufficient sleep to investigate slow and fast spindles separately, or enough SWS to test whether a different pattern of results was observed during SWS. This limitation is not unexpected given the difficulty in obtaining long, consolidated bouts of sleep in an MRI environment. As scanner technology becomes more comfortable and less noisy, future studies may succeed at obtaining longer duration sleep in the MRI simultaneously with EEG. Taken together, animal and recent human neuroimaging studies, including the current study, support the involvement of thalamocortical, striatal, and cerebellar circuitry in spindle generation. However, these findings do not directly investigate the functional correlates of spontaneous spindle-related brain activation, discussed in the following section.

### Association Between Spindle-Related Activation and Reasoning Ability

The results of the current study identified, for the first time, that brain activations recruited during spontaneous spindle events were specifically associated with Reasoning abilities, but not STM or Verbal abilities, including the thalamus, the PFC (i.e., the ACC/MCC), the bilateral putamen, the cerebellum, and the precuneus. Thus, suggesting that interindividual differences in the extent of activations in cortico–thalamic–striatal circuitry time-locked spindles are related to individual cognitive strengths, and in particular, Reasoning abilities. The extent of these activations does not relate to other cognitive abilities, e.g., STM or Verbal abilities. Given the widespread nature and heterogeneity of the brain regions activated, further investigation to explore the network dynamics (e.g., using functional connectivity analyses) is warranted. This may reveal network hubs of communication, for which the strength of connectivity with other areas might provide further insight into the relationship between spindles and intellectual abilities. It is important to note that the results of this study are entirely correlational, and the possibility remains that the observed correlation can be explained by other confounding or unrelated factors. Moreover, the directionality of this relationship cannot be determined from the results of this study. Future studies employing similar techniques in cases where spindle activity is abnormal and where specific cognitive deficits occur such as Autism (Limoges et al., 2013), learning disabilities (Shibagaki et al., 1982), and in schizophrenia (Wamsley et al., 2012) may provide additional insight into the neural basis of the relationship between spindles and Reasoning abilities. However, this study is an important first step in investigating the functional significance of spindle-related activations in young, healthy populations.

The network of brain regions identified here, activated during spindles, correlated with Reasoning abilities are convergent with the extant literature. For example, thalamo–cortical circuitry (e.g., thalamus and the PFC region) is implicated in modulation of cognitive performance, such as memory, executive functioning, and attention (Van Der Werf et al., 2000; Van der Werf et al., 2003; Blair, 2006; Mitchell and Chakraborty, 2013; Ferguson and Gao, 2015). Similar to the current study, thalamic activations have been observed while solving fluid intelligence tasks, including problem solving (Fangmeier et al., 2006), reasoning tasks (Melrose et al., 2007), and particularly, inductive reasoning (Jia et al., 2011; Liang et al., 2014). Other neuroanatomical studies (Bohlken et al., 2014) found that thalamic volume was significantly correlated with general intellectual functioning. In addition, at least one study identified structural and functional abnormalities in the thalamus in adults with reduced intellectual functioning who experienced prenatal exposure to alcohol (Clark et al., 2000). Similarly, a large body of literature has identified the role of the PFC in fluid intelligence and Reasoning (Baker et al., 1996; Waltz et al., 1999; Duncan, 2000; Gray et al., 2003; Coricelli and Nagel, 2009). For example, patients with damage to the PFC exhibited a selective and catastrophic deficit for both deductive and inductive reasoning tasks (Waltz et al., 1999). In addition, Gray et al. (2003) found that individuals with higher fluid intelligence have greater activations in the PFC. Coricelli and Nagel (2009) have shown that Reasoning abilities correlate with neural activity in the medial PFC. Additionally, at least one neuroanatomical MRI study employing voxel-based morphometry has revealed a positive correlation between gray matter intensity in the medial PFC and Reasoning abilities assessed by Cattell’s Culture Fair Intelligence Test, and also the WAIS-R (Gong et al., 2005). In addition, several studies have observed robust activations in the basal ganglia for Reasoning-related tasks compared to other cognitive tasks, including the caudate nucleus, putamen, and globus pallidus (Melrose et al., 2007; Rodriguez-Moreno and Hirsch, 2009). Specifically, Sandman et al. (2014) has reported that the morphometry of the putamen was associated with performance on Reasoning-related subtests of the WAIS including block design, matrix Reasoning, and perceptual index in preadolescent children. Although overlooked in some studies, the cerebellum is activated during the deductive reasoning processing (Goel et al., 2000; Goel and Dolan, 2004). Thus, taken together, suggesting that integrity and functioning of these regions (e.g., thalamus, PFC, striatum, cerebellum) are required for intact Reasoning abilities, however, this possibility remains to be explicitly investigated. Our results suggest that individuals with greater spindle-related activation of this circuitry is associated with greater Reasoning abilities. Thus, spindles may be an important marker of Reasoning abilities, and a window into understanding the interindividual differences in the activation patterns of neural substrates related to specific cognitive abilities.

The clinical significance and applications of the relationship between spindles and cognitive abilities is yet to be realized. Interestingly, spindle production is reduced with age (Carrier et al., 2001, 2011; Lafortune et al., 2012; Martin et al., 2013; Fogel et al., 2014; Fogel et al., 2017b), and abnormal in developmental disorders, such as Autism (Limoges et al., 2005), learning disabilities (Shibagaki et al., 1982), and in schizophrenia (Wamsley et al., 2012). Deficient or dysfunctional spindle generation has been found to be associated with compromised intellectual functioning. More specifically, it has been suggested that deficient gating mechanisms of thalamocortical circuitry (Bixler and Rhodes, 1968) may explain abnormal spindle production in children with mental disability (Gibbs and Gibbs, 1962; Shibagaki and Kiyono, 1983). Moreover, the present study is an important first step which may lead to the development of novel interventions utilizing spindle-enhancing neuromodulatory techniques (e.g., neurofeedback, transcranial direct current stimulation, pharmacological techniques) to improve daytime cognitive performance and explore the physiological mechanisms which support the function of sleep for memory and cognitive performance. Thus, such an approach could target cognitive deficits, in cases where spindle production is abnormal such as in learning disabilities (Gibbs and Gibbs, 1962; Shibagaki and Kiyono, 1983), below normal cognitive functioning (Fogel and Smith, 2011), normal, healthy aging (Carrier et al., 2011; Fogel et al., 2014, 2017b), developmental disorders (Limoges et al., 2005), and in schizophrenia (Wamsley et al., 2012). A better understanding of the neural basis of the relationship between spindles and cognitive abilities may ultimately help to better understand biological basis of normal and abnormal cognitive functioning in healthy individuals and neurological conditions. This may eventually lead to novel interventions to precisely target cases where spindle production is abnormal or non-optimal.

However, there are limitations of the current study worth mentioning. First, sleep was recorded only for a short time at the beginning of the night, in an MRI environment. Thus, we would expect that the architecture of sleep and spindle parameters are not perfectly comparable to a whole night of natural, nocturnal sleep in a normal sleeping environment. It should be mentioned that the density of sleep spindles in the current study was higher than some previous EEG studies. There are several possible reasons for this discrepancy. First, studies have shown that spindle density is highest near the start of the first NREM cycle in a night (Zeitlhofer et al., 1997; Purcell et al., 2017) and the density of sleep spindles is decreased with the deepening of sleep (Steriade and Amzica, 1998; De Gennaro et al., 2000; Himanen et al., 2002). In the current study, we recorded sleep only at the beginning of the night, and under conditions where deep sleep is less likely. Thus, spindle density would be expected to be higher than whole-night studies under normal conditions. Moreover, the continuous noise from the MRI scanner throughout the sleep session may have had an unavoidable impact on spindle production. A number of previous studies have shown that sleep spindles can protect sleep from external stimuli (Peters and Jones, 1991; Elton et al., 1997; Cote et al., 2000; Vyazovskiy et al., 2004; Dang-Vu et al., 2010; Schabus et al., 2012). For example, Dang-Vu et al. (2010) have demonstrated that individuals who generate a greater number of sleep spindles maintain sleep better in the face of disruptive stimuli. Therefore, it is possible that participants who slept better generated a greater number of spindles. Finally, sleep in an MR environment is likely more comparable to daytime napping recordings (i.e., light, fragmented sleep). Accordingly, the density of sleep spindles reported in the current study is comparable to a previous daytime napping study using the same spindle detection method (Albouy et al., 2013). Second, while the majority of subjects (*N* = 20) achieved SWS, there is however, enormous inter-subject variability in terms of the actual duration (spindle number range: 1–752, mean = 133.35, Std = 177.93). Furthermore, only 12 subjects had more than 30 spindle events (spindle number range: 42–752, mean = 218, Std = 189.18) during slow wave sleep, which is the minimum reasonable number necessary to carry out the analyses. As a result of these issues, we were not confident of the results for SWS only. In addition, we do not have any specific *a priori* hypotheses about SWS spindles in particular. Therefore, we did not separate the stage 2 sleep and slow wave sleep in the current study. Finally, the goal of the current study was to investigate the event-related functional brain activations in the BOLD signal time course that occur during spindle events. Therefore, similar to previous EEG–fMRI sleep studies investigating spindle-related brain activations (Schabus et al., 2007; Andrade et al., 2011; Fogel et al., 2017a), spindles at the midline derivation (re; Fz, Cz, Pz) were detected to identify the onsets of spindle events given that the topographical distribution of visually scored and automatically detected sleep spindles is prevalent on central leads, especially the centroparietal midline derivation (McCormick et al., 1997; Zeitlhofer et al., 1997). Also, it has been reported that most spindles are detected simultaneously from multiple channels, whereby spindles tend to co-occur at midline channels more than half the time (Hagler et al., 2018). However, it is important to note that in spite of the prevalence and concurrence patterns of spindles maximal at central derivation spindles, there is evidence that sleep spindles also occur locally (Andrillon et al., 2011; Nir et al., 2011; Piantoni et al., 2016; Hagler et al., 2018). Local spindles occurring in isolation may be associated with specific aspects of cognitive functions, such as working memory (Buchmann et al., 2014), memory consolidation (Johnson et al., 2010), and global cognitive integration (Manoach et al., 2016). While beyond the scope of the objectives of the current study, deep-source EEG localization techniques could provide corroborative or converging evidence in regards to whether EEG sources correspond to BOLD brain activations time-locked to sleep spindles from a single scalp location (e.g., Boutin et al., 2018). This may shed light on the functional significance of local vs. global spindle events.

In summary, we investigated the spindle-related neural substrates that support cognitive strengths and weaknesses. There are considerable interindividual differences in sleep spindles, which are very trait-like (Silverstein and Levy, 1976; Gaillard and Blois, 1981). While the neural circuitry and generating mechanisms of spindles are well-understood, the neurophysiological basis of the relationship between spindles and cognitive abilities remains to be fully elucidated. Our results show for the first time the neuroanatomical functional correlates of the relationship between sleep spindles and specific intellectual abilities. In particularly, our study found that the extent of the brain activations time-locked to sleep spindles were correlated with interindividual differences in Reasoning, but not Verbal or STM abilities. These findings provide new insights to understand the function of sleep spindles.

## Author Contributions

SF and AO designed the study and supervised the research. SF, ZF, and LR carried out the research and collected the data. ZF, SF, and LR contributed to data analyses. ZF, SF, and LR prepared the figures and wrote the manuscript. All authors discussed the results and commented on the manuscript.

## Conflict of Interest Statement

The authors declare that the research was conducted in the absence of any commercial or financial relationships that could be construed as a potential conflict of interest.

## Footnotes

1 https://www.cambridgebrainsciences.com/science/tasks

2 https://github.com/jadesjardins/vised_marks

3 github.com/stuartfogel/detect_spindles

4 http://www.fil.ion.ucl.ac.uk/spm/software/spm8/

5 http://marsbar.sourceforge.net/
