# Supplementary material for: Brain Activation Time-Locked to Sleep Spindles Associated With Human Cognitive Abilities
Source: Front Neurosci. 2019 Feb 6;13:46. doi: 10.3389/fnins.2019.00046 (PMC6372948; doi:10.3389/fnins.2019.00046)
Supplement: Supplementary file 1 [file Data_Sheet_1.docx]

Supplementary Material


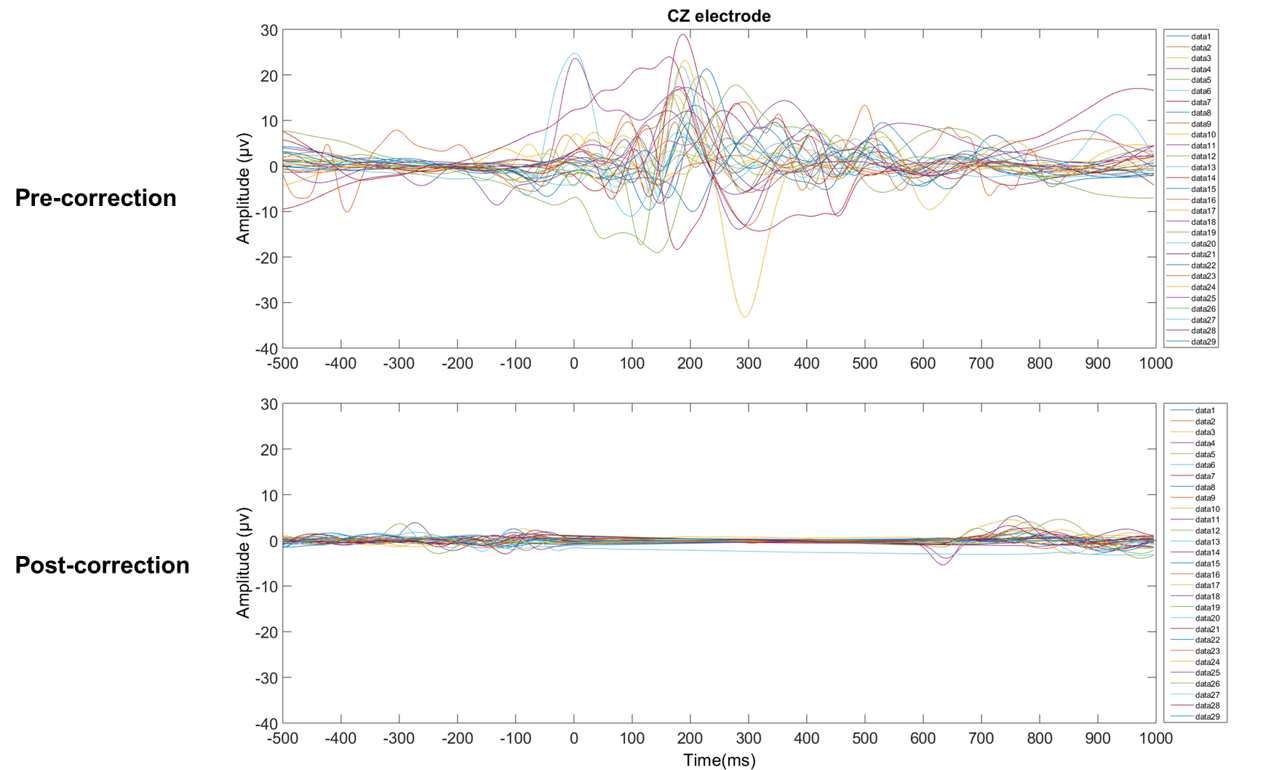


**Figure S1.** Amplitudes of individual residual artifacts time-locked to the r-peaks before and after artifact correction.


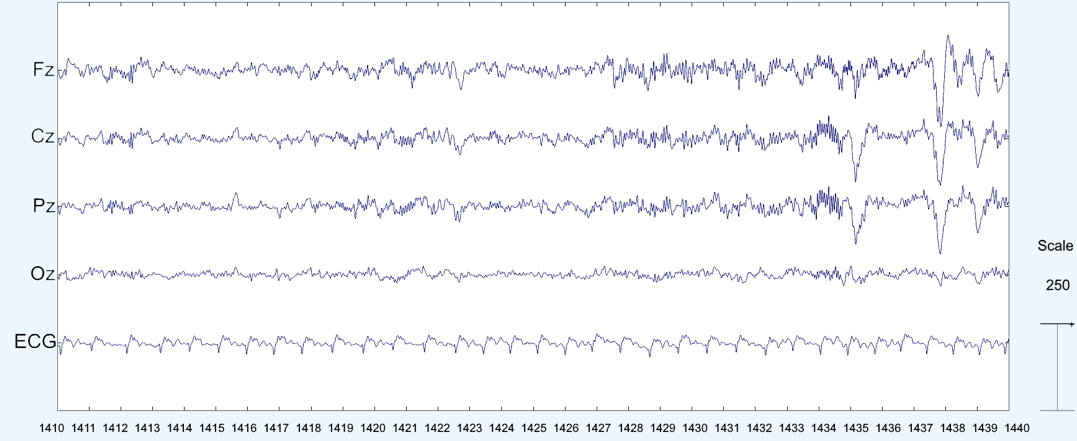


**Figure S2. Screenshot for EEG tracing of one single subject in NREM sleep.** Residual gradient and BCG artifacts visibly removed from the EEG tracing after artifact correction.

**Partial correlation analyses between subtests of Reasoning subscale and spindle amplitude**

To further explore which subtest score (i.e. deductive reasoning, spatial rotation, feature match, spatial planning, and polygons) from the Reasoning subscale was correlated with spindle amplitude, partial correlation analysis revealed that deductive reasoning, spatial planning and polygons were all significantly correlated with spindle amplitude (p < 0.05), while spatial rotation and spatial planning were marginally correlated (p <0.10) with spindle amplitude (Supplemental Table S1).

| Table S1. Partial correlations between spindle amplitude and subtests scores of Reassoning abilities | | | | | |
| --- | --- | --- | --- | --- | --- |
|  | Deductive Reasoning | spatial  rotation | feature  match | spatial  planning | polygons |
| Spindle amplitude | r = 0.516 | r = 0.345 | r = 0.331 | r = 0.444 | r = 0.445 |
|  | p = 0.006* | p = 0.078 | p = 0.092 | p = 0.020* | p = 0.020* |
| Note: the r value indicates the partial correlation coefficients. Statistically significant results indicated by an asterisk (*) at p < .05. | | | | | |

**Partial correlation analyses between subtests of Reasoning subscale and brain activation time-locked to spindle**

| Table S2 Partial correlation between brain activation time-lockerd to spindles and subtests scores of Reassoning abilities | | | | | |
| --- | --- | --- | --- | --- | --- |
|  | Deductive Reasoning | spatial  rotation | feature  match | spatial  planning | Polygons |
| Thalamus activation | r = 0.409 | r = 0.454 | r = 0.320 | r = 0.580 | r = 0.525 |
|  | p = 0.034* | p = 0.017* | p = 0.104 | p = 0.002** | p = 0.005* |
| ACC/MCC activation | r = 0.394 | r = 0.568 | r = 0.272 | r = 0.362 | r = 0.430 |
|  | p = 0.042* | p = 0.002* | p = 0.169 | p = 0.064 | p = 0.025* |
| Putamen activation | r = 0.385 | r = 0.538 | r = 0.300 | r = 0.410 | r = 0.598 |
|  | p = 0.047* | p = 0.004* | p = 0.129 | p = 0.034* | p = 0.001* |
| Note: the r value indicates the partial correlation coefficients. Statistically significant results indicated by an asterisk (*) at p < .05. | | | | | |

The same partial correlation analyses were conducted between the five subtests scores and the spindle-related brain activation in the three main regions of interest (i.e., thalamus, ACC/MCC, putamen). As shown in Supplemental Table S2, all subtests were correlated with brain activations time-locked to spindles in thalamus and putamen (p < 0.05) except for feature match. In addition, the ACC/MCC activation was significantly correlated with deductive reasoning, spatial rotation, and polygons (p < 0.05), and marginally correlated with spatial planning (p = 0.06).

**Descriptions of the 12 subtests of Cambridge Brain Sciences Tests (CBS)**

The 12 tasks are adapted from well-known, well-established paradigms from the cognitive neuroscience literature. Using factor analysis, three cognition domains (i.e. Reasoning, Short term memory, Verbal) are derived from these 12 subtests (Table S3).

| **Table S3.** Three cognition domains and 12 subtests of Cambridge Brain Sciences Tests | | |
| --- | --- | --- |
| **Cognition domains** | **Subtest Task** | **Reference** |
| Reasoning | deductive reasoning | (Cattell, 1940) |
|  | spatial rotation | (Silverman et al., 2000) |
|  | feature match | (Treisman & Gelade, 1980) |
|  | spatial planning | (Shallice, 1982), |
|  | Polygons | (Folstein et al.,1975) |
| Short term memory | visuospatial working memory | (Inoue & Matsuzawa, 2007) |
|  | spatial span | (Corsi, 1972) |
|  | paired associates | (Gould et al.,2006), |
|  | Self-order search | (Collins et al., 1998) |
| Verbal | verbal reasoning | (Baddeley, 1968) |
|  | color–word remapping | (Stroop, 1935) |
|  | digit span | (Wechsler, 1981) |

All tests are available for evaluation at <https://www.cambridgebrainsciences.com/science/tasks>. Screenshot and description of each task are shown below:


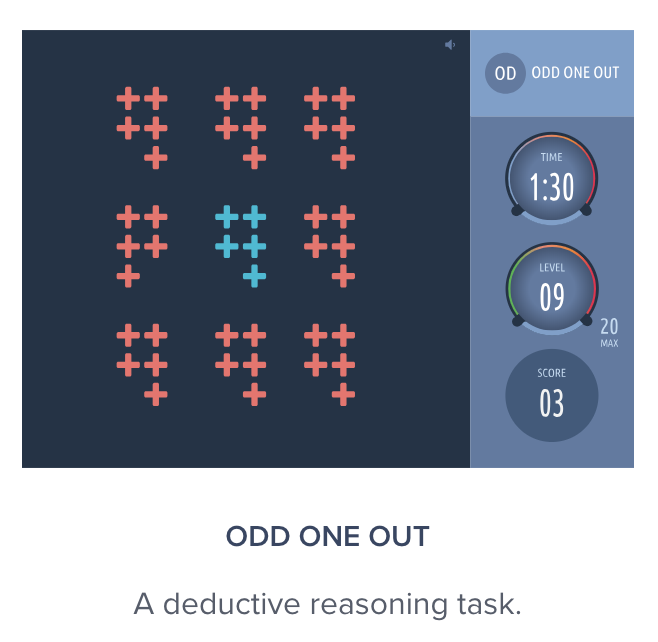


1. **Odd one out assesses deductive reasoning,** which is the core cognitive ability to apply rules to information in order to arrive at a logical conclusion. Odd one out requires reasoning about the features of several shapes to deduce the one shape that does not fit in with the rest. Nine sets of shapes appear on the screen, different from each other in color, shape, and number. Participant must pay close attention to how the shapes differ from each other, and point out the one shape that is most different from the rest. Participants have 90 seconds to solve as many problems as possible. In some cases, like when there is only one red shape, the answer is obvious. However, the task gets more difficult with each correct answer, and harder puzzles require comparing several different features at the same time.

**
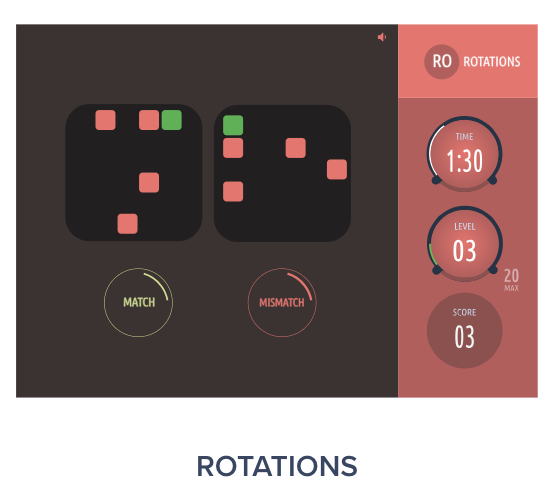
**

1. **Spatial rotation** is a function of visual representation in the brain. Effectively manipulating mental representations of objects allow people to make valid conclusions about what objects are and where they belong. Two groups of colored squares (each with N squares) are displayed on the screen, each filled with red and green squares. The participant must determine if the boxes would be identical if one of them could be rotated. More squares are added each time the participant answers correctly, increasing the difficulty. Participants have 90 seconds to solve as many problems as possible. A correct response increases the final score by N, and the subsequent trial has groups of N+1 squares. If the response is incorrect, the total score decreases by N, and next trial has groups of N-1 squares.


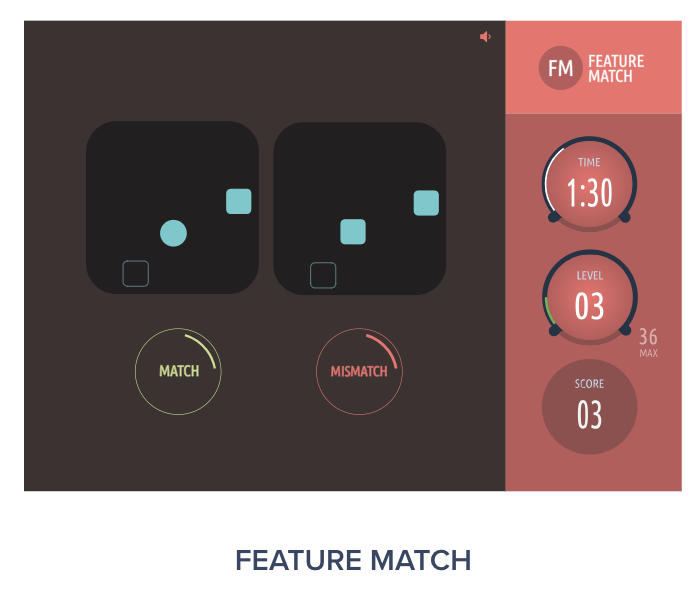


1. **Feature Match.** A task measuring the ability to muster mental resources to focus and monitor for a specific stimulus or difference. Identifying similarities and differences is an important real-life skill that is put to the test in this difficult version of “spot the difference”. Two boxes appear on the screen, each containing an array of abstract shapes. Participants must determine if the boxes are identical or different and click the appropriate button. Difficulty adjusts to the participants’ performance, ensuring the task is consistently challenging. Participants have 90 seconds to solve as many problems as possible. A correct response increases the final score by N, and the subsequent trial has groups of N+1 items. If the response is incorrect, the total score decreases by N, and next trial has groups of N-1 items.


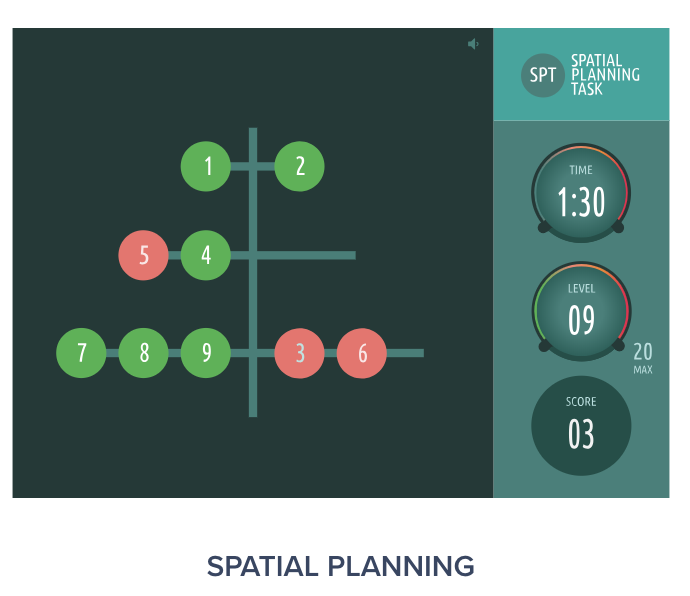


1. **Spatial Planning**. Planning is a fundamental property of intelligent behaviour, which is based on the Tower of London Task. Spatial Planning assesses participants’ ability to act with forethought and sequence behaviour in an orderly fashion to reach specific goals. A tree-shaped frame appears on the screen with 9 numbered balls slotted onto the branches. The participant must rearrange the balls so that they are slotted onto the branches in numerical order, in as few moves as possible. Participants have 3 minutes to solve as many problems as possible. Puzzles get more difficult as the participants gets correct answers.

**
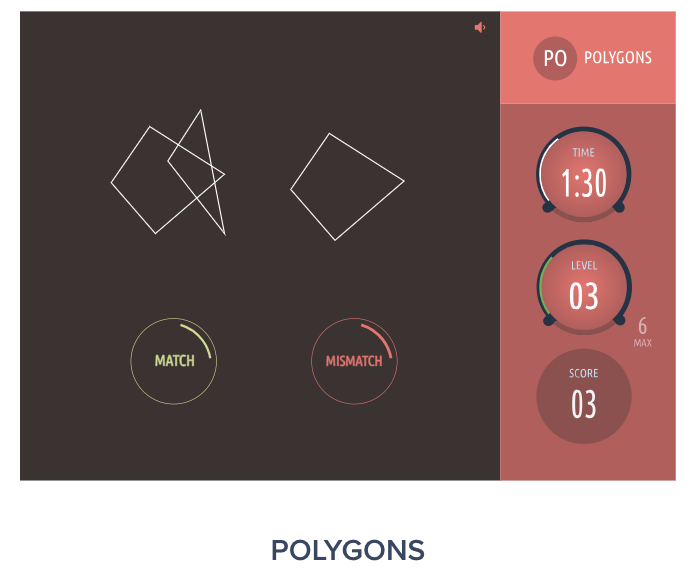
**

1. **Polygons** assesses visuospatial processing, which is the ability to effectively interpret visual information, such as complex visual stimuli and relationships between objects. Polygons challenges the participant’s proficiency in picking out subtle differences between shapes. Two panels appear. One contains two overlapping shapes, and one contains just one shape. The participant must determine if the single shape is identical to one of the overlapping shapes, or if it is subtly different than both shapes. Puzzles get more difficult with every correct answer. Participants have 90 seconds to solve as many problems as possible. An incorrect response decreases the total score by the difficulty level, and the next trial will be slightly easier.


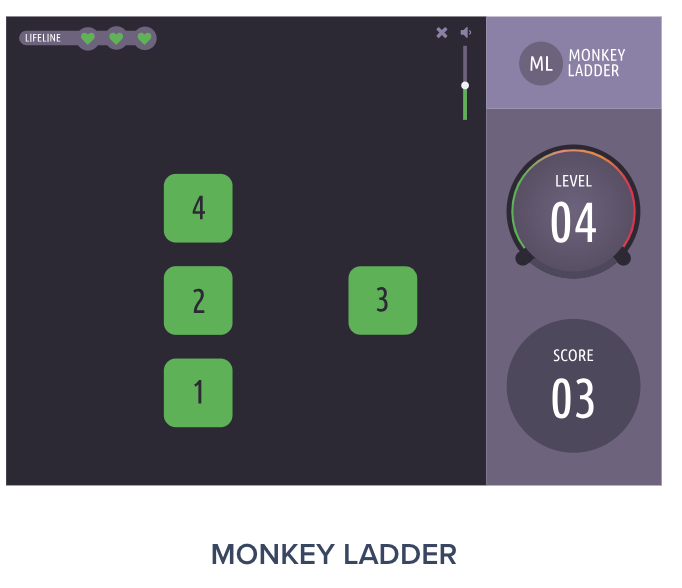


1. **Monkey ladder** assesses visuospatial working memory, which is the ability to not only hold information in memory, but manipulate or update it based on changing circumstances. Monkey Ladder requires storing numbers and their locations, then translating that memory into a series of movements in space. Boxes appear at different locations on the screen, each containing a number. Participant must try to remember which numbers appear in which box. After a short time, the numbers disappear, and the participant clicks on the boxes in numerical sequence. Difficulty adjusts to the participants’ performance, and performance is indicated by the average number of boxes correctly remembered. The test finishes after 3 errors, and the resulting score is the length of the longest sequence successfully remembered.


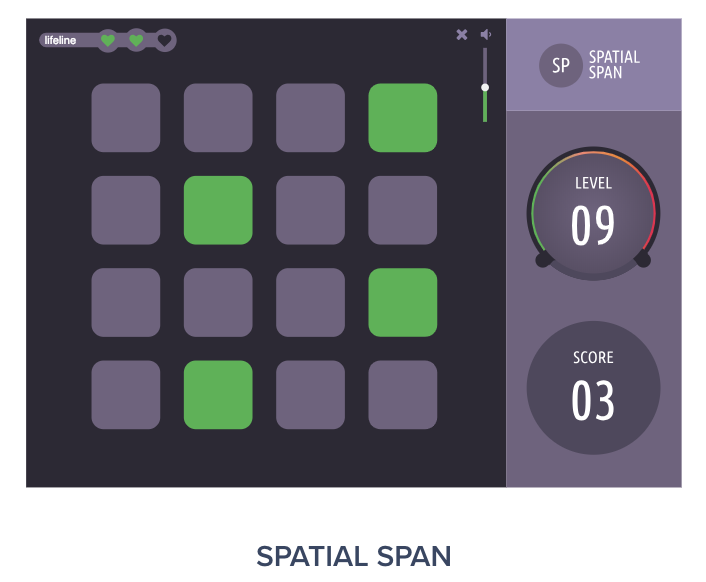


1. Spatial span based on Corsi Block Tapping Task, assesses Spatial short-term memory, which is the cognitive system allowing for temporary storage of spatial information. Spatial Span challenges the participant’s ability to remember the relationships between objects in space, as opposed to verbally rehearsing items in specific order, which relies on verbal short-term memory. A grid of boxes appears on the screen. The participant’s job is to pay attention when the boxes begin flashing in sequence, then click the boxes in the same sequence. If correct, the next sequence will be one box longer. The test finishes after 3 errors. The score is the length of the longest sequence successfully remembered.


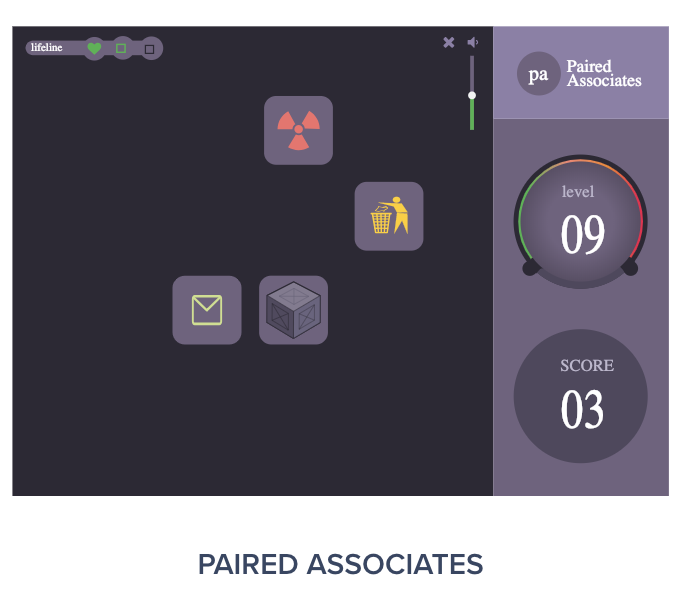


1. **Paired associates** assess episodic memory, which is the ability to remember and recall specific events, paired with the context in which they occurred. Participants are required to remember which objects they previously saw, along with the location where they were seen. A set of boxes appear on the screen. They will open, one after the other, revealing the objects inside. The participant must remember which object appeared in which box. Next, one at a time, objects appear in the center of the screen, and participants must point out which box each object was located in. The test ends after three errors. The participant’s score is the maximum number of pairs successfully remembered. Participants have 90 seconds to solve as many problems as possible.


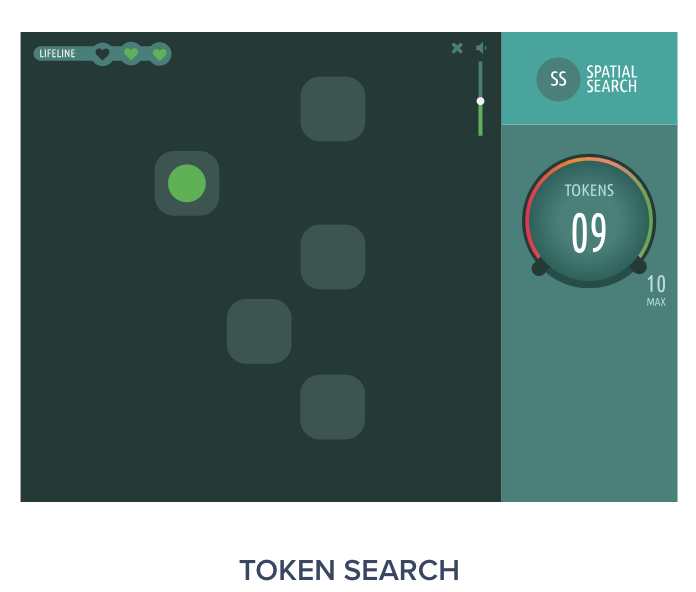


1. **Self-order search or Token search** assess the working memory, which is the ability to temporarily hold information in memory, and manipulate it based on changing circumstances or demands. In Token Search, participants need to maintain and update an ongoing representation of previous searches in a self-directed task. Several boxes appear on the screen. The participant must click boxes to search them, looking for a token. The process repeats when a token is found, but the box where a token was previously found cannot be searched again, requiring ongoing updates to the representation of the boxes in memory. Correctly finding a token in every box will present a new puzzle with more boxes. The test finishes after three errors. The resulting score is the maximum level completed.


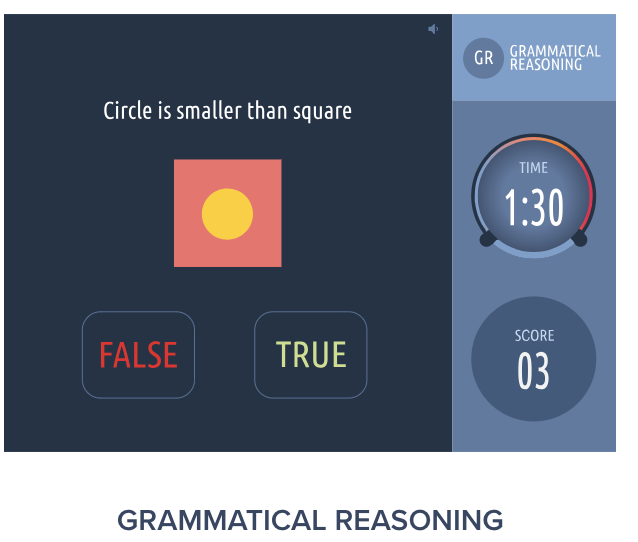


1. Grammatical reasoning assessed the Verbal reasoning, which is the ability to quickly understand and make valid conclusions about concepts expressed in words. While language comes naturally to most people, understanding complex sentences with multiple negative statements is consistently challenging. A statement appears at the top of the screen, and two objects underneath. The participant’s task is to reason about the relationships among the objects and determine if the statement is true or false. Responding quickly and accurately is required for high scores. Participants have 90 seconds to solve as many problems as possible. A correct response increases the total score by one point, and an incorrect response decrease the score by one point.


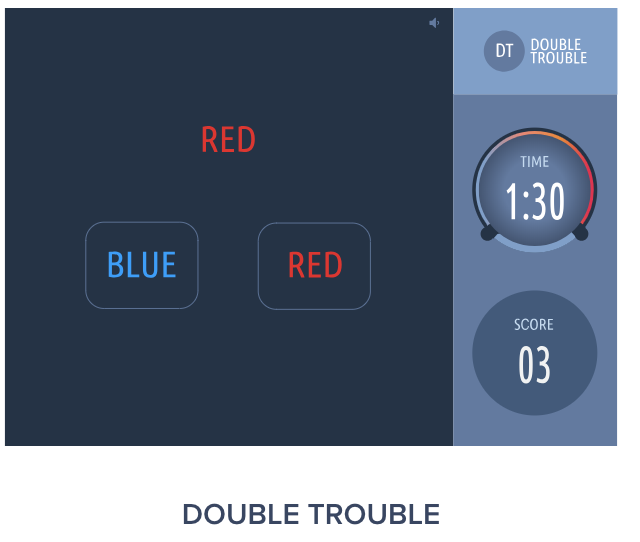


1. **Color-word remapping or Double trouble** (based upon the stroop task) assesses response inhibition, which is the ability to concentrate on relevant information to make an appropriate response, even when distracting information or interference is present. It is a key component of concentration. Three words appear on the screen: one at the top and two at the bottom. The user's job is to click on the word at the bottom that correctly describes the colour of the word at the top—for example, if the word at the top says “BLUE” but is written in red, the participant must inhibit the tendency to read what the word says, and instead click the word “RED.” Participants have 90 seconds to solve as many problems as possible. A correct response increases the total score by one point, and an incorrect response decrease the score by one point.


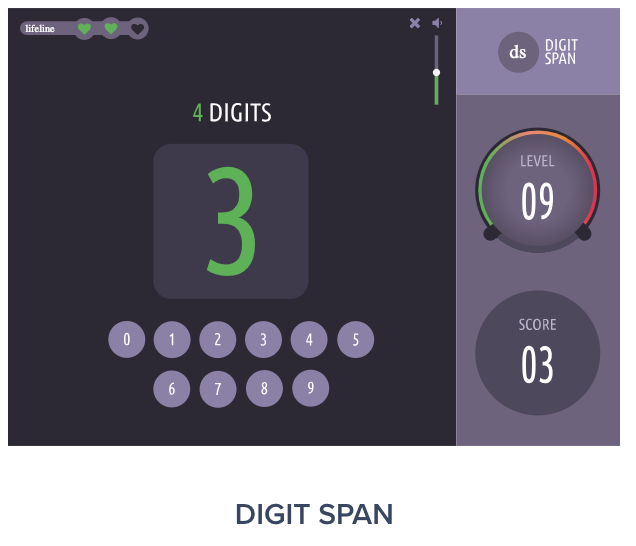


1. **Digit Span** measures verbal short-term memory, defined as the system that allows for temporary storage of information, and is crucial in everyday tasks such as remembering a telephone number or understanding long sentences. Digit Span involves numbers, but performance is indicative of verbal short-term memory, because it requires dealing with items in a specific order, as opposed to spatial short-term memory. A sequence of numbers appears on the screen, one at a time. At the sound of the beep, users click the numbers in the same order. the test ends after 3 mistakes. The resulting score is the length of the longest digit sequence successfully remembered.
